# The Antirheumatic Drug Gold, a Coin With Two Faces: AU(I) and AU(III). Desired and Undesired Effects on the Immune System

**DOI:** 10.1155/MBD.1994.483

**Published:** 1994

**Authors:** Kazuo Takahashi, Peter Griem, Carsten Goebel, Jose Gonzalez, Ernst Gleichmann

**Affiliations:** 1 Division of Immunology Medical Institute of Environmental Hygiene Heinrich Heine University Düsseldorf Auf'm Hennekamp 50 Düsseldorf D-40225 Germany

## Abstract

Three new findings are reviewed that help to understand the mechanisms of action of antirheumatic Au(I) drugs, such as disodium aurothiomalate (Na_2_Au(I)TM): (i) We found that Na_2_Au(I)TM selectively inhibits T cell receptor (TCR)-mediated antigen recognition by murine CD^4+^ T cell hybridomas specific for antigenic peptides containing at least two cysteine residues. Presumably, Au(I) acts as a chelating agent forming linear complexes (Cys-Au(I)-Cys) which prevent correct antigen-processing and/or peptide recognition by the TCR. (ii) We were able to show that Au(I) is oxidized to Au(III) in phagocytic cells, such as macrophages. Because Au(III) is re-reduced to Au(I) this may introduce an Au(I)/Au(III) redox system into phagocytes which scavenges reactive oxygen species, such as OCl^-^ and inactivates lysosomal enzymes. (iii) Pretreatment with Au(III) of a model protein antigen, bovine ribonuclease A (RNase A), induced novel antigenic determinants recognized by CD^4+^ T lymphocytes. Analysis of the fine specificity of these ‘Au(III)-specific’ T cells revealed that they react to RNase peptides that are not presented to T cells when the native protein, i.e., not treated with Au(III), is used as antigen. The T cell recognition of these cryptic peptides did not require the presence of gold. This finding has important implications for understanding the pathogenesis of allergic and autoimmune responses induced by Au(I) drugs. Taken together, our findings indicate that Au(I) and Au(III) each exert specific effects on several distinct components of macrophages and the subsequent activation of T cells; these effects may explain both the desired anti-inflammatory and the adverse effects of antirheumatic gold drugs.


### THE ANTIRHEUMATIC DRUG GOLD, A COIN WITH TWO FACES: AU(I) AND AU(III). DESIRED AND UNDESIRED EFFECTS ON THE IMMUNE SYSTEM

Kazuo Takahashi, Peter Griem, Carsten Goebel, Jose Gonzalez, and Ernst Gleichmann

Division of Immunology, Medical Institute of Environmental Hygiene at Heinrich Heine University Düsseldorf, Auf'm Hennekamp 50, D-40225 Düsseldorf, Germany

### ABSTRACT

Three new findings are reviewed that help to understand the mechanisms of action of antirheumatic Au(I) drugs, such as disodium aurothiomalate ($Na_2Au(I)TM$): (i) We found that $Na_2Au(I)TM$ selectively inhibits T cell receptor (TCR)-mediated antigen recognition by murine $CD4^+$ T cell hybridomas specific for antigenic peptides containing at least two cysteine residues. Presumably, Au(I) acts as a chelating agent forming linear complexes (Cys-Au(I)-Cys) which prevent correct antigen-processing and/or peptide recognition by the TCR. (ii) We were able to show that Au(I) is oxidized to Au(III) in phagocytic cells, such as macrophages. Because Au(III) is re-reduced to Au(I) this may introduce an Au(I)/Au(III) redox system into phagocytes which scavenges reactive oxygen species, such as $OCl^-$ and inactivates lysosomal enzymes. (iii) Pretreatment with Au(III) of a model protein antigen, bovine ribonuclease A (RNase A), induced novel antigenic determinants recognized by $CD4^+$ T lymphocytes. Analysis of the fine specificity of these 'Au(III)-specific' T cells revealed that they react to RNase peptides that are not presented to T cells when the native protein, i.e., not treated with Au(III), is used as antigen. The T cell recognition of these cryptic peptides did not require the presence of gold. This finding has important implications for understanding the pathogenesis of allergic and autoimmune responses induced by Au(I) drugs. Taken together, our findings indicate that Au(I) and Au(III) each exert specific effects on several distinct components of macrophages and the subsequent activation of T cells; these effects may explain both the desired anti-inflammatory and the adverse effects of antirheumatic gold drugs.

**Keywords:** antirheumatic gold drugs, $CD4^+$ T lymphocytes, adverse immune reactions, immunopharmacology, immunotoxicology

---

*Financially supported by the program Research on Autoimmunity, grant no. 01 KD 89030, from the Federal Ministry of Research and Technology, Bonn, Federal Republic of Germany and by grant no. CT 29-0316 "In vitro Immunotoxicology" from the BIOTECH program of the EU, Brussels, Belgium.
**Author to whom correspondence should be adressed.

### INTRODUCTION

All antirheumatic gold drugs, such as disodium aurothiomalate ($Na_2Au(I)TM$) contain gold in the oxidation state +1. The mechanism of action of gold drugs is unknown, but they are effective in the treatment of rheumatoid arthritis. With respect to both their therapeutic and adverse effects gold drugs have been termed 'slow-acting' because there is a lag period of several weeks to months before they become effective (1, 2). Eventually adverse immune reactions necessitate discontinuation of gold therapy in up to one-third of patients (3-5). The immunopathologic alterations developing under gold therapy (6) are summarized in Table 1. Although antirheumatic gold drugs have been successfully used for almost six decades, the mechanism of action underlying their beneficial and their adverse effects are still largely unknown. This represents a challenge to both immunopharmacology and immunotoxicology. Here, we shall review recent results from our laboratory that help to understand the mechanism of action of these compounds.

**Table 1:** Adverse immunologic side-effects of gold(I) drugs, such as $Na_2Au(I)TM$, and their genetic association

| Pathological alterations | Man | Mouse |
|---|---|---|
| Dermatitis | + | |
| Stomatitis | + | |
| Alveolitis | + | |
| Eosinophilia | + | |
| Spleen and lymph node enlargement | | + |
| IC glomerulonephritis | + | + |
| IgG and IgE increase | + | + |
| Antinuclear autoantibodies | + | + |
| Thrombopenia | + | |
| Granulocytopenia | + | |
| IgG and IgA decrease | + | |
| Aplastic anemia | + | |
| Genetic association: | | |
| MHC | + | + |
| Slow sulfoxidation status | + | |

Since many years it has been known that gold is stored and accumulated in mononuclear phagocytes, especially their lysosomes or lysosome-like organelles (7-10). In vitro, Au(I) can inhibit lysosomal enzymes, such as ß-glucuronidase and acid phosphatase (11), which are known to participate in inflammatory reactions in the joints. In recent years, however, it has become clear that lysosomal proteases play a crucial role in antigen-processing which takes place in lysosomes and related organelles of antigen-presenting cells (APC) (cf. Fig. 1). The term 'antigen-processing' refers to the enzymatic cleavage of protein antigens into short peptides which then can bind to MHC class II molecules. Subsequently, the peptide-MHC complexes are transported to the surface of APC, where they are recognized by the T cell receptor (TCR) of $CD4^+$ T lymphocytes. It is important to know that APC cannot distinguish self proteins from foreign proteins so that they process both types of protein. Because T cells are tolerant of or anergic to those self peptides that are efficiently processed and presented, they fail to be activated by them. By contrast, the presentation of foreign peptides or self peptides not normally presented, i.e., cryptic peptides, will activate T cells and thus trigger an immune response. In our view, the accumulation and long-term storage of gold could well be relevant for its mechanism of action. Any hypothesis trying to explain the mechanism of action of gold drugs should try to incorporate the accumulation of gold in the lysosomes of phagocytic cells.

### GOLD KINETICS IN HUMANS AND MICE UNDER LONG-TERM TREATMENT WITH Au(I) COMPOUNDS

Following the administration of antirheumatic Au(I) drugs to patients with rheumatoid arthritis, numerous investigations, applying a variety of different techniques, were undertaken to measure the concentration of gold in plasma and tissues (reviewed in reference 12). It was consistently found that 80 - 90 % of the gold detectable in plasma is bound to serum albumin (13-15) and that gold accumulates in kidney, liver, and spleen (16-20). Using morphological techniques for the examination of these organs, gold was found to be stored preferentially in lysosomes or lysosome-like organelles of phagocytic cells (7-10). Ghadially (7), who used electron X-ray analysis to describe such lysosomal bodies containing gold, introduced the term 'aurosomes' for these organelles. Other investigators who showed the uptake and storage of gold in phagocytes used electron microscopy and autometallography, respectively (10, 21, 22). In all these studies, however, gold concentrations were only determined after cessation of treatment, never during ongoing treatment. Moreover, if the kinetics of gold in the body were studied at all, these measurements were confined to but a few organs and points in time.

As no systematic pharmacokinetic studies had been performed in animals or men to determine gold tissue concentrations under chronic treatment, our group performed such a study in mice using chronic treatment with $Na_2Au(I)TM$ and atomic absorption spectrometry (AAS) (12). Mice of three different inbred strains, A.SW, C57BL/6, and DBA/2, received weekly intramuscular injections of 22.5 mg $Na_2Au(I)TM$ / kg body weight over a maximal period of 14 weeks. It should be noted that the dosage used exceeds that given to rheumatoid arthritis patients by a factor of at least 30. This difference in dosage was chosen because of the greater metabolic activity in mice compared to men and because previous investigations had shown that this dosage induced immunologic side-effects in mice similar in those seen in men (6, 23-25).

The results obtained by Tonn et al. (12) indicated that the tissue gold levels showed significant differences both between strains and, in a time-dependent manner, within a given strain and tissue, respectively. In all three tissues studied, especially the spleen, strain DBA/2 contained lower gold levels than strains C57BL/6 and A.SW. Whereas the latter two strains are known to be susceptible to the adverse immunologic side-effects of $Na_2Au(I)TM$, strain DBA/2 is resistant (6, 23-25). An observation made in all three mouse strains was that the concentration of gold in the spleen, an organ of the immune system, steadily increased until week 10 or 12. Throughout the observation period the resistant strain DBA/2 showed a lower splenic gold concentration than the two susceptible mouse strains. Interestingly, of the three splenic cell types studied, T and B cells of all three strains failed to contain measurable concentrations of gold whereas splenic macrophages contained high levels. Similarly, high levels of gold were detectable in peritoneal cells, known to be rich in macrophages. These findings support the view (18, 21, 26) that the impairment of lymphocyte activation induced by gold compounds (1, 27, 28) is due to an effect of gold on accessory cells, such as macrophages, rather than to a direct effect on lymphocytes (29).

The high gold concentration in macrophages is consistent with reports of altered monocyte/macrophage functions resulting from exposure to gold salts in vitro (30-33) and in vivo (34, 35). Severals authors (7, 9, 18, 21, 36) have described the accumulation and storage of gold in lysosomes or lysosome-like cellular bodies of macrophages, and it is conceivable that these organelles are the primary anatomical site of the pharmacologic and toxicologic action of gold drugs at the subcellular level.

### Au(I) INTERFERES WITH ANTIGEN-PROCESSING IN MACROPHAGES AND THE PRESENTATION OF IMMUNOGENIC PEPTIDES CONTAINING TWO OR MORE CYSTEINE RESIDUES

A number of findings suggest that the pharmacological mechanism of Au(I) drugs directly or indirectly involves an effect on T lymphocytes. $Na_2Au(I)TM$ was found to inhibit the proliferation of human T cells to IL-2 in vitro at concentrations higher than 15 µM (27). Proliferation of human T cells induced by stimulation with phorbol myristate acetate (PMA) and anti-CD3 monoclonal antibody (mAb) was inhibited by $Na_2Au(I)TM$ concentrations higher than 30 µM (37). $Na_2Au(I)TM$ inhibited protein kinase C (PKC) activity, but had no effect on the generation of inositol 1,4,5 trisphosphate ($IP_3$) (37). These findings suggest that $Na_2Au(I)TM$ might suppress T cell activation by interfering with transmembrane signalling. Inhibition of Concanavalin A (Con A)-induced T cell proliferation by $Na_2Au(I)TM$, in contrast, seemed to be mediated indirectly by an effect on the costimulatory capacity of monocytes (29). An effect on MHC class II-bearing cells that can function as APC was also implied by the observations that Au(I) bound to MHC molecules (38). These results led to the hypothesis that gold might alter antigen presentation or T cell recognition of presented MHC-peptide complexes (39).

Based on these findings we investigated the effect of $Na_2Au(I)TM$ on the processing and presentation of protein antigens to T cells. Using a panel of murine $CD4^+$ T cell hybridomas specific for bovine RNase A, bovine insulin, hen egg lysozyme, apamin, and cobra (Naja nigricollis) α toxin, respectively, we demonstrated that $Na_2Au(I)TM$ inhibited the interleukin-2 (IL-2) response of certain hybridoma clones (P. Griem, K. Takahashi, H. Kalbacher and E. Gleichmann, manuscript submitted). This effect was observed at $Na_2Au(I)TM$ concentrations that were smaller than 10 µM and, thus, were not cytotoxic for T cells and were in the same range as tissue gold concentrations of rheumatoid arthritis patients (20, 40), which for instance reach 10 - 50 µM in serum and about 10 - 100 µM in synovial fluid. The inhibition was not caused by a cytotoxic effect on the antigen presenting cells or on the affected T-hybridoma cells. Disodium thiomalate alone had no effect on the T cell reaction. $Na_2Au(I)TM$ affected only the antigen-induced but not the anti-CD3 monoclonal antibody-induced IL-2 release. We concluded that Au(I) could inhibit activation of certain T cell clones by either interfering with antigen-processing or interaction of the presented peptide-MHC complex with the TCR.

The T cell recognition of four peptides that contained two or more cysteine residues could be inhibited by $Na_2Au(I)TM$, whereas of five peptides with one or no cysteine residue, only one peptide was weakly affected. Because Au(I) preferentially forms linear complexes with two thiol ligands (20), we propose that Au(I) binds to free thiol groups of the antigen. The binding of Au(I) to two thiol groups on the same peptide, forming a chelate complex, should be much more stable than a ternary complex (41) formed by one peptide thiol group, Au(I), and another thiol group, e.g. from free cysteine or glutathione. Chelate complex formation could explain why the inhibitory effect of $Na_2Au(I)TM$ requires the presence of two thiol groups in the antigenic peptide. We propose that the observed inhibition of T cell activation by $Na_2Au(I)TM$ might be mediated by binding of Au(I) to thiol groups of MHC-embedded peptides at the cell surface or to partially processed antigen inside lysosomes, where Au(I) is known to accumulate (12, 40, 42, 43). This complex formation might lead to steric hindrance of TCR-MHC interaction or interference with further processing of antigen and binding of antigenic peptides to MHC molecules.

The selective immunosuppressive effect of $Na_2Au(I)TM$ in vitro described here might contribute to the beneficial effect of gold compounds in the treatment of rheumatoid arthritis, because it matches the requirements (40) for rational explanation of clinical findings (26, 40, 44). The observed inter-patient variability of the beneficial gold effect is not unexpected because remission should occur only when the major arthritogenic peptide(s) contain(s) at least two cysteine residues, which is unlikely to be the case in all patients. Intramolecular and intermolecular determinant spreading (45) during the disease process may be responsible for the fact that sustained benefit is rare and often variable over time. The slow onset of clinical benefit during gold therapy could imply that it takes some time and a certain tissue concentration before Au(I) can reduce the density of presented arthritogenic peptides below the minimal density required for T cell activation (46). Finally, the proposed selective effect of Au(I) drugs on certain peptides would be in line with the lack of general immunosuppression during gold therapy (40, 47). Whether the proposed mechanism really contributes to the therapeutic effect of Au(I) compounds in rheumatoid arthritis will probably not be answered until the relevant autoantigens are identified. It will then be possible to answer the question whether Au(I) drugs have a greater beneficial effect in patients in which the major arthritiogenic peptides contain two or more cysteine residues.

### BIOOXIDATION OF Au(I) TO Au(III) IN VIVO

At least some of the immunopathologic side-effects seen in man also develop in susceptible mouse strains treated with weekly intramuscular injections of $Na_2Au(I)TM$ (Table 1). Equimolar injections of the carrier disodium thiomalate ($Na_2TM$), by contrast, fail to induce any of these anormalities (6, 23, 48). As in humans, genes linked to the MHC as well as non-MHC genes are involved in the immunologic susceptibility of mice to $Na_2Au(I)TM$ (23). A strain-dependent susceptibility to treatment with an Au(I) drug has also been demonstrated in rats. Brown Norway rats developed an increased serum IgE level, proteinuria, and autoantibodies to the glomerular basement membrane, whereas Lewis rats failed to do so (49).

Several lines of evidence suggest that T lymphocytes play a central role in the development of immunopathologic reactions to gold(I) drugs (50). When this hypothesis was tested in susceptible mouse strains (6), a perplexing result was obtained. The gold(I) drug $Na_2Au(I)TM$, although a potent inducer of adverse immune reactions upon weekly intramuscular injections, failed to elicit the expected T cell reaction in the direct popliteal lymph node assay (PLNA). By contrast, equimolar amounts of Au(III) salts readily induced a T cell-dependent PLN reaction. The PLNA is an in vivo test system for measuring specific T cell reactions toward chemicals of low molecular weight (6, 51-53).

To explain this discrepancy the hypothesis has been proposed that in vivo the Au(I) of the slow-acting drug $Na_2Au(I)TM$ is gradually oxidized to Au(III), and that it is the latter which elicits T cell sensitization and thus initiates the GVH-like side-effects. This was experimentally tested by Schuhmann et al. (6) using the adoptive transfer

PLNA. The results of this experiment demonstrated that, indeed, weekly intramuscular injections of Au(I), given in the form of $Na_2Au(I)TM$, sensitized T cells neither to Au(I) nor to $Na_2TM$ but to Au(III), even though the latter had never been administered to the T cell donors.

In the adoptive transfer PLNA (6, 51-53) the test compound, such as $Na_2Au(I)TM$, is first administered to donor animals according to a schedule that is relevant to human use of this compound. (In humans, $Na_2Au(I)TM$ is given by repeated intramuscular injections.) Subsequently, T cells of the donor animals are isolated from their spleens and injected subcutaneously into one hind foot pad of a syngeneic recipient animal. The recipient serves merely as an indicator of reactivity of the transferred T cells because, in addition to the donor T cells, the test compound or one of its metabolites is injected into the same foot pad. As in the direct PLNA, lymph node weight, cell number, or cell proliferation is measured 6 days later. By means of this assay system it can be asked whether T cells of the donor animals have been sensitized to the test compound or a metabolite. If in their new environment the donor T cells re-encounter the antigen to which they have been sensitized previously, they mount a secondary immune response. The latter manifests itself by quantitative and qualitative changes of lymphoid cells in the draining PLN of the recipient. The dose of test antigen used in the adoptive transfer PLNA is always below the minimal dose required for inducing a primary PLN response. Thus, a primary PLN response of the recipient animal is avoided, but anamnestic responses of the donor T cells transferred can be detected.

On the basis of their findings obtained with the adoptive transfer PLNA, Schumann et al. (6) concluded that the adverse immune effects of Au(I) salts are in fact caused by Au(III) which must be generated in vivo through oxidation of Au(I). In both animals and humans, gold is known to be accumulated and stored in the lysosomes of monocytes and macrophages (7, 54, 55). Here, Au(I) might be oxidized to Au(III) by reactive oxygen species, such as HOCl (Fig. 1). In fact, in a cell-free system in vitro, which contained the HOCl-generating factors $H_2O_2$, $Cl^-$, and myeloperoxidase (MPO), Au(III) was generated from $Na_2Au(I)TM$ (56; and paper by C.F.Shaw III et al., this volume). Furthermore, Au(III), other than Au(I), can irreversibly oxidize proteins (20, 57) and thus may be considered a reactive intermediate metabolite. Altered self protein(s), in turn, if presented by MHC molecules on the surface of macrophages, could elicit GVH-like reactions by T cells. In the presence of protein, Au(III) exists only for a few minutes (20, 58) because immediately after its generation Au(III) oxidizes proteins and is thus itself reduced to Au(I) again.

In the study of Schuhmann et al. (6) sensitized T cells were used as a probe to detect alterations induced by Au(III), a short-lived reactive intermediate metabolite that in biological samples is not or is only detectable with difficulty by physicochemical methods. Because T cells are known not to react to free metal ions, such as Au(III), but react to MHC-embedded peptides (59), the observed T cell reactions cannot have been directed to Au(III) itself, but to self protein(s)/peptide(s) altered by this highly reactive heavy metal ion. In other words, the observed T cell reactions presumably were not directed to the metabolite but to its footprints left on self protein(s)/peptide(s) or on MHC molecules themselves (Fig. 1).

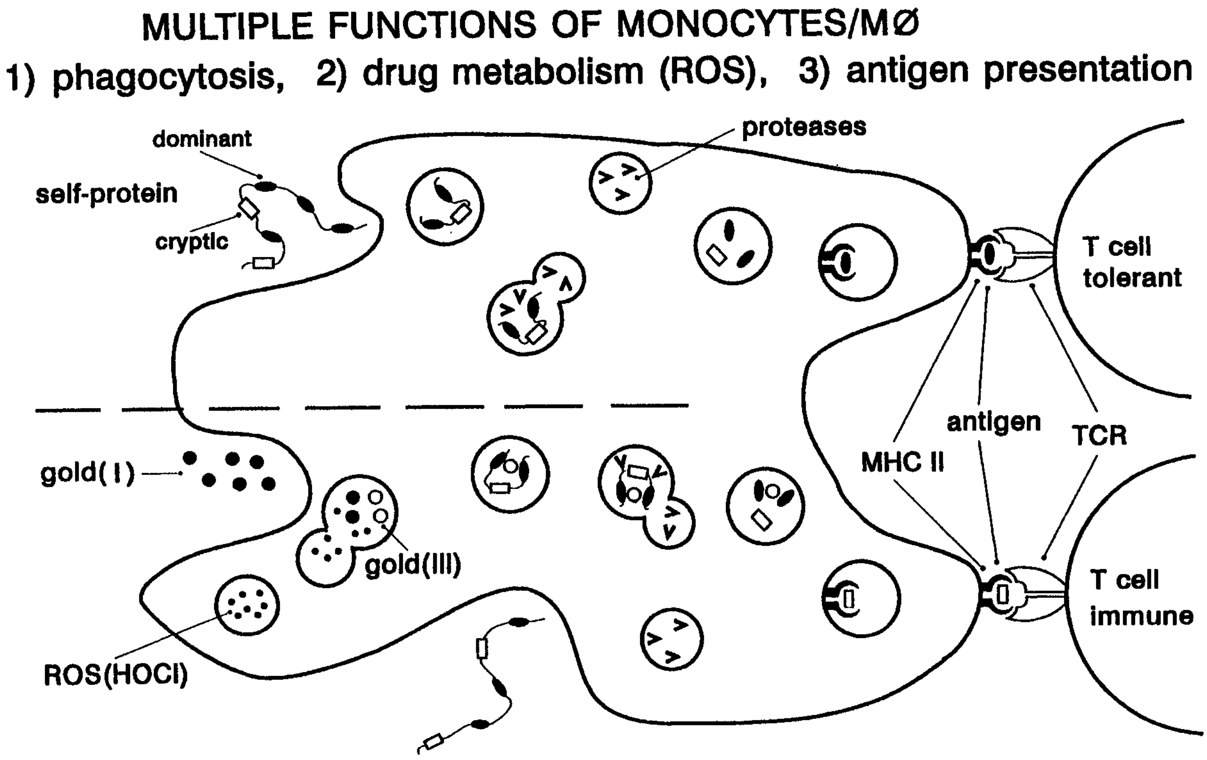

**Figure 1.** Monocytes and macrophages are involved in the formation of reactive drug metabolites which than may interfer with the physiological presentation of self proteins. The upper part of the figure shows the processing and presentation of a self protein. Like microorganisms that have invaded the body, self proteins are taken up by macrophages. The proteins are partially degraded into peptides by lysosomal proteases. Of the peptides generated the ones that bind best to MHC class II molecules are transported to the cell surface, where the peptide-MHC complexes are presented to T cells that recognize these with their T cell receptor (TCR). Only certain peptides (termed dominant peptides) are efficiently cut out from the protein and presented on the cell surface. Due to the induction of tolerance during T cell development, presentation of these peptides does not lead to T cell activation. The lower part of the figure shows the oxidation of Au(I) to Au(III) by reactive oxygen species (ROS), like hypochloric acid (HOCl), in the lysosomal compartment. Oxidation of the self protein by the reactive intermediate metabolite Au(III) may induce an alteration in its processing leading to the presentation of peptides that are not normally presented (termed cryptic peptides). Since these peptides are new to the immune system, no T cell tolerance to them exists and an immune response is initiated.

### THE BIOOXIDATION OF Au(I) TO Au(III) TAKES PLACE IN PHAGOCYTIC CELLS

Recently our group was able to provide direct experimental evidence for the concept (Fig. 1) that biooxidation of Au(I) to Au(III) takes place in phagocytic cells, such as monocytes and macrophages. T cells of mice were primed to Au(III) either by weekly intramuscular injections of Au(I) in the form of $Na_2$Au(I)TM, or by three subcutaneous injections of HAu(III)$Cl_4$. Using the adoptive transfer PLNA, the transferred T cells of these animals were found to react in an anamnestic fashion not only to the Au(III) salt, but also to homogenized macrophages pretreated with $Na_2$Au(I)TM. The macrophages used were either activated syngeneic macrophages that had been incubated with $Na_2$Au(I)TM in vitro or peritoneal cells obtained from syngeneic donors that had received weekly intramuscular injections of $Na_2$Au(I)TM for 12 weeks (C. Goebel, M. Kubicka-Muranyi, T. Tonn, J. Gonzalez, and E. Gleichmann, manuscript submitted).

In humans who had been treated with Au(I) drugs for rheumatoid arthritis and had subsequently developed dermatitits, anamnestic T cells responses to Au(III) were also observed (39, 60, 61; and T. Tonn, unpublished results). Verwilghen et al. (61) tested peripheral blood lymphocytes of 17 patients with acute gold dermatitis in the lymphocyte transformation test in vitro, and found that the cells of 13 of them reacted to Au(III), but not to $Na_2$Au(I)TM or other heavy metal salts.

While the results of Verwilghen et al. (61) clearly reflect the differential immunogenicity of Au(I) and Au(III) salts and thus confirm in humans the findings obtained in mice (6), the picture is not quite as clearcut in all investigations. In a few patients a positive reaction to both Au(III) and Au(I) was observed (T. Tonn, unpublished results). Here, it can be argued that the putative reaction to Au(I) was in fact directed towards Au(III) that was generated by monocytes through oxidation of Au(I) during the 1-week culture period. This explanation, however, can only be applied with difficulty to the findings of Romagnoli et al. (39). They found that two T cell clones obtained from the same patient reacted to both Au(I) and Au(III) even when the antigen-presenting cells (Epstein-Barr virus-transformed autologous B cells) were glutaraldehyde-fixed. It is generally agreed that glutaraldehyde fixation inactivates enzyme activities in cells (62, 63); if true, this would also pertain to the enzymes involved in the oxidation of Au(I) to Au(III). A possible explanation for the inability of the two cell clones to distinguish between Au(I) and Au(III) could be that the Au(I) salt induces a conformational change of MHC class II molecules (64). Conceivably, the two T cell clones studied by Romagnoli et al. (39) were specific for class II molecules thus changed. Because Au(III) is rapidly reduced to Au(I) by protein, it may have caused identical reactions as Au(I) in this particular case.

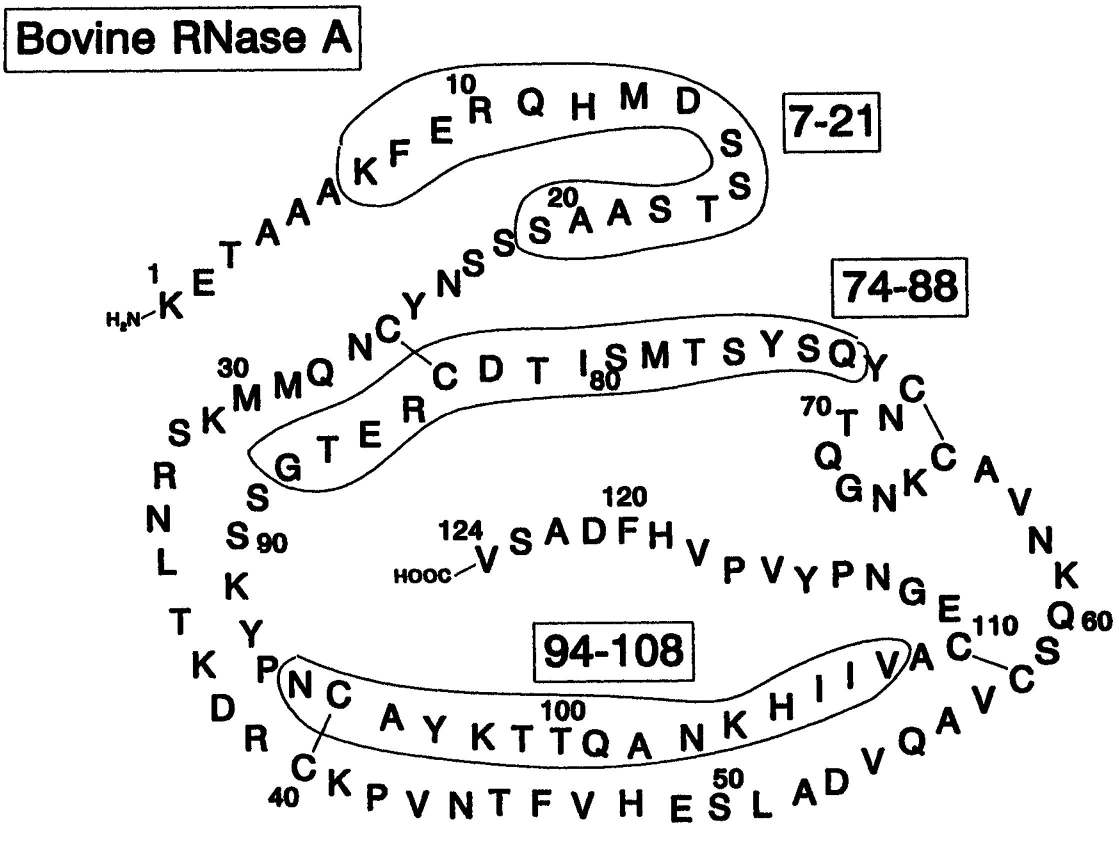

**Figure 2.** Dominant and cryptic peptides of bovine ribonuclease A (RNase A). Immunization of C57BL/6 mice with native RNase A leads to activation of T cells that are specific for peptide 74-88. When mice were immunized with RNase A that had been oxidized with Au(III) (58), additional T cell specificities were found. T cell clones that reacted to RNase/Au(III) but not to native RNase, were found to be specific for peptide 7-21 or peptide 94-108. Au(III) addition was not required for the recognition of the peptides but alteration of the protein by Au(III) was necessary for efficient cleavage of these peptides from the protein during antigen-processing.

### Au(III) INDUCES THE PRESENTATION OF CRYPTIC PEPTIDES

As the self proteins altered by Au(III) have not yet been identified, we used bovine RNase A as a model antigen and oxidized it by pretreatment with Au(III) at pH 2, as described by Isab and Sadler (58). Mice were immunized against RNase/Au(III), and $CD4^+$ T cell hybridomas specifically recognizing RNase/Au(III), but not native RNase, were established (P. Griem, K. Beyer, and E. Gleichmann, manuscript submitted). By using a set of overlapping peptides of bovine RNase A, each 15 amino acids long, we were able to identify two peptides recognized by these T cell hybridomas (Fig. 2). These peptides are not presented when APC are pulsed with native RNase, but only after pulsing them with RNase/Au(III). Moreover, the presence of gold was not necessary for the regognition of these peptides by the RNase/Au(III)-specific T cell clones. Hence, denaturing RNase by Au(III) led to presentation of cryptic peptides of bovine RNase A. Conceivably, the same happens with self protein exposed to Au(III) leading to the presentation of cryptic self peptides. By definition, T cells fail to be tolerant to cryptic self peptides so that adverse immune reactions may develop.

**Au(I)/Au(III) redox system:**

1) Au(I) → Au(III)

Au(I) is phagocytosed and induces ROS in phagolysosomes → ROS, such as HOCl, have been scavenged

2) Au(III) → Au(I)

native protein → protein denatured by Au(III)

| Au(I) | Au(III) |
|---|---|
| **ANTI-INFLAMMATORY EFFECTS** | |
| -scavenges ROS<br>-inactivates thiol-containing lysosomal enzymes<br>-chelates arthritogenic peptides containing two or more cysteines and thus blocks their recognition by TCR | -denatures lysosomal proteins, such as elastase and MPO<br>-denatures arthritogenic peptides and/or MHC II molecules |
| **ADVERSE EFFECTS** | |
| -possibly creates new epitopes on self-proteins by the chelating effect | -denatures self-proteins and thus leads to presentation by MHC II molecules of cryptic peptides that activate specific $CD4^+$ T cells |

**Figure 3.** Hypothetical mechanisms for anti-inflammatory and adverse effects of Au(I) and Au(III).

Arguments for phagocytic cells as potential sites for the generation of reactive drug metabolites (66) were summarized above. Regarding the actual immunogenicity of drug metabolites thus generated, the findings obtained with Au(I)/Au(III) were the first to give experimental support to this important concept. Au(III), however, is a somewhat unusual intermediate whose generation in the body has been demonstrated only in the indirect fashion outlined above. In contrast, the production by phagocytes of reactive metabolites of procainamide (66-69) is generally accepted. Therefore, we recently started to perform similar experiments with procainamide and its reactive metabolites using the same experimental approach as with Au(I)/Au(III).

### POSSIBLE IMPLICATIONS OF THE BIOOXIDATION OF Au(I) TO Au(III)

Generation of the highly reactive intermediate Au(III) could also be relevant with respect to the unknown pharmacological mechanism of action of Au(I) drugs. Three anti-inflammatory mechanisms of Au(III) may be envisaged (Fig. 3). Firstly, the generation in phagocytic cells of Au(III) from Au(I) scavenges reactive oxygen species. Secondly, as already mentioned, Au(III) is a highly reactive chemical that irreversibly denatures protein (57). The candidate proteins to be denatured by Au(III) are those nearest to the site of oxidation of Au(I) to Au(III). Presumably these are the proteins located in the phago-lysosomes of phagocytic cells (60, 65), such as lysosomal enzymes, which are known to enhance inflammation when released from cells. Thirdly, Au(III) may interfere with those lysosomal enzymes involved in antigen-processing or may directly alter MHC molecules. Both mechanisms could result in reduced production or presentation of arthritogenic (self) peptides. These anti-inflammatory effects of gold should be enhanced and prolonged by the fact that Au(III) is very short-lived in the presence of protein because it rapidly oxidizes protein and is re-reduced to Au(I) (20, 58). In fact, as pointed out by C.F. Shaw III (personal communication, 1991), on the basis of the findings of Schuhmann et al. (6) gold therapy may introduce a Au(I)/Au(III) redox system into phagocytic cells (Fig. 3). If this view is correct, the three anti-inflammatory actions of Au(I)/Au(III) described above would be effective over a prolonged period of time.

ACKNOWLEDGEMENTS

We thank E. Tosse GmbH and Company (Hamburg, Germany) for a gift of aurothiomalic acid in the form of Tauredon™.

### REFERENCES

1. Dillard, C. J. & Tappel, A. L. (1986) Med. Hypotheses **20**, 407-420.

2. Lipsky, P. E. (1984) Agents Actions Suppl. **14**, 181-204.

3. Davis, P. (1979) J. Rheumatol. **6**, 18-23.

4. Gran, J. T., Husby, G. & Thorsby, E. (1983) Ann. Rheum. Dis. **42**, 63-71.

5. Lockie, L. M. & Smith, D. M. (1985) Sem. Arthritis Rheum. **14**, 238-246.

6. Schuhmann, D., Kubicka-Muranyi, M., Mirtscheva, J., Günther, J., Kind, P. & Gleichmann, E. (1990) J. Immunol. **145**, 2132-2139.

7. Ghadially, F. N. (1979) J. Rheumatol. **6**, 45-50.

8. Gottlieb, N. L., Smith, P. M. & Smith, E. M. (1972) Arthritis Rheum. **15**, 16.

9. Graudal, H., Möller-Madsen, B. & Danscher, G. (1988) Z. Rheumatol. **47**, 347-350.

10. Danscher, G., Hansen, H. J. & Möller-Madsen, B. (1984) Histochemistry **81**, 283-285.

11. Lee, M. T., Ahmed, T., Haddad, R. & Friedman, M. E. (1989) J. Enzym. Inhib. **3**, 35-47.

12. Tonn, T., Goebel, C., Wilhelm, M. & Gleichmann, E. (1994) Br. J. Rheumatol. **33**, 724-730.

13. McQueen, E. G. & Dykes, P. W. (1994) Ann. Rheum. Dis. **28**, 437.
14. Danpure, C. J., Fyfe, D. A. & Gumpel, J. M. (1979) Ann. Rheum. Dis. **38**, 364.
15. Egila, J., Littlejohn, D., Smith, W. E. & Sturrock, R. D. (1992) J. Pharm. Biomed. Anal. **10**, 639-644.
16. Swartz, H. A., Christian, J. E. & Andrews, F. N. (1960) Am. J. Physiol. **199**, 67.
17. Sugawa-Katayama, Y., Koishi, H. & Danbara, H. (1975) J. Nutrition **104**, 957.
18. Sharma, R. P. & McQueen, E. G. (1979) Clin. Exp. Pharmacol. Physiol. **6**, 561.
19. Lewis, A. J. & Walz, D. T. (1982) in Progress in medicinal chemistry, eds. Ellis, G. P. & West, G. B. (Elsevier Biomedical Press, New York), pp. 1-58.
20. Shaw, C. F. (1979) Inorg. Perspect. Biol. Med. **2**, 287-355.
21. Lawson, K. J., Danpure, C. J. & Fyfe, D. A. (1977) Biochem. Pharmacol. **26**, 2417-2426.
22. Möller-Madsen, B., Mogensen, S. C. & Danscher, G. (1984) Exp. Mol. Pathol. **40**, 148-154.
23. Robinson, C. J. G., Balazs, T. & Egorov, I. K. (1986) Toxicol. Appl. Pharmacol. **86**, 159-169.
24. Mirtscheva, J., Hallmann, B., Stark, M. & Gleichmann, E. (1986) Arch. Pharmacol. **334**, 171.
25. Pietsch, P., Vohr, H. W., Degitz, K. & Gleichmann, E. (1989) Int. Arch. Allergy Appl. Immunol. **90**, 47-53.
26. Kavanaugh, A. F. & Lipsky, P. E. (1992) in Inflammation, eds. Gallin, J. I., Goldstein, I. M. & Snyderman, R. (Raven Press, New York), pp. 1083-1101.
27. Wolf, R. E. & Hall, V. C. (1988) Arthritis Rheum. **31**, 176-181.
28. Hashimoto, K., Whithurst, C. E., Matsubara, T., Hirohata, K. & Lipsky, P. E. (1992) J. Clin. Invest. **89**, 1839-1848.
29. Lipsky, P. E. & Ziff, M. (1977) J. Clin. Invest. **59**, 455-466.
30. Littman, B. H. & Hall, R. E. (1985) Arthritis Rheum. **28**, 1384-1392.
31. Ugai, K., Ziff, M. & Lipsky, P. E. (1979) Arthritis Rheum. **22**, 1352-1360.
32. Scheinberg, M. A., Santos, L. M. B. & Finkelstein, A. E. (1982) J. Rheumatol. **9**, 366-369.
33. Seitz, M., Dewald, B., Ceska, M., Gerber, N. & Baggiolini, M. (1992) Rheumatol. Int. **12**, 159-164.
34. Farahat, M. N. M. R., Yanni, G., Poston, R. & Panayi, G. S. (1992) EULAR Congress Reports **1**, 8.
35. Laib, J. E., Shaw, C. F., Petering, D. H., Eidsness, M. K., Elder, R. C. & Garvey, J. S. (1985) Biochemistry **24**, 1977-1986.
36. Christensen, M. M., Danscher, G., Ellermann Eriksen, S., Schionning, J. D. & Rungby, J. (1992) Histochemistry **97**, 207-211.
37. Hashimoto, K., Whitehurst, C. E. & Lipsky, P. E. (1994) J. Rheumatol. **21**, 1020-1026.
38. Sinigaglia, F. (1994) J. Invest. Dermatol. **102**, 398-401.
39. Romagnoli, P., Spinas, G. A. & Sinigaglia, F. (1992) J. Clin. Invest. **89**, 254-258.
40. Champion, G. D., Graham, G. G. & Ziegler, J. B. (1990) Baillieres. Clin. Rheumatol. **4**, 491-534.
41. Shaw, C. F. (1989) Comments Inorg. Chem. **8**, 233-267.
42. Möller-Madsen, B., Morgensen, S. C. & Danscher, G. (1984) Exp. Mol. Pathol. **40**, 148-154.

43. Elder, R. C., Eidsness, M. K., Heeg, M. J., Tepperman, K. G., Shaw, C. F. & Schaeffer, N. (1983) ACS Symposium Series **209**, 385-400.
44. Cash, J. M. & Klippel, J. H. (1994) N. Engl. J. Med. **330**, 1368-1375.
45. Kaufman, D. L., Clare Salzler, M., Tian, J. D., Forsthuber, T., Ting, G. S. P., Robinson, P., Atkinson, M. A., Sercarz, E. E., Tobin, A. J. & Lehmann, P. V. (1993) Nature **366**, 69-72.
46. Harding, C. V. & Unanue, E. R. (1990) Nature **346**, 574-576.
47. Lipsky, P. E. (1988) in Inflammation: Basic Principles and Clinical Correlates, eds. Gallin, J. I., Goldstein, I. M. & Snyderman, R. (Raven Press, Ltd., New York), pp. 897-910.
48. Cush, J. J., Lipsky, P. E., Postlethwaite, A. E., Schrohenloher, R. E., Saway, A. & Koopman, W. J. (1990) Arthritis Rheum. **33**, 19-28.
49. Tournade, H., Guery, J. C., Pasquier, R., Nochy, D., Hinglais, N., Guilbert, B., Druet, P. & Pelletier, L. (1991) Nephrol. Dial. Transplant. **6**, 621-630.
50. Gleichmann, E., Pals, S. T., Rolink, A. G., Radaszkiewicz, T. & Gleichmann, H. (1984) Immunol. Today **5**, 324-332.
51. Gleichmann, E., Kind, P., Schuppe, H. C. & Merk, H. (1990) in Immunotoxicity of Metals and Immunotoxicology, eds. Dayan, A. D., Hertel, R. F., Heseltine, E., Kazantzis, G., Smith, E. M. & Van der Venne, M. T. (Plenum Press, New York), pp. 139-152.
52. Klinkhammer, C., Popowa, P. & Gleichmann, H. (1988) Diabetes **37**, 74-80.
53. Kubicka-Muranyi, M., Behmer, O., Uhrberg, M., Klonowski, H., Bister, J. & Gleichmann, E. (1993) Int. J. Immunopharmacol. **15**, 151-161.
54. Brown, D. H. & Smith, W. E. (1980) Chem. Soc. Rev. **2**, 217-241.
55. Jakobson, E., Andreasen, A., Graudal, H. & Danscher, G. (1989) Scand. J. Rheumatol. **18**, 161-164.
56. Beverly, B. & Couri, D. (1987) Fed. Proc. **46**, 854.
57. Witkiewicz, P. & Shaw, C. F. (1981) J. C. S. Res. Comm. 1111-1114.
58. Isab, A. A. & Sadler, P. J. (1977) Biochim. Biophys. Acta **492**, 322-330.
59. Rothbard, J. B. & Gefter, M. L. (1991) Annu. Rev. Immunol. **9**, 527-565.
60. Gleichmann, E., Kubicka-Muranyi, M., Kind, P., Goldermann, R., Goerz, G., Merk, H. & Rau, R. (1991) Rheumatol. Int. **11**, 219-220.
61. Verwilghen, J., Kingsley, G. H., Gambling, L. & Panayi, G. S. (1992) Arthritis Rheum. **35**, 1413-1418.
62. Delovitch, T. L., Semple, J. W. & Philips, M. L. (1988) Immunol. Today **9**, 216-219.
63. Shimonkevitz, R., Kappler, J., Marrack, P. & Grey, H. (1983) J. Exp. Med. **158**, 303-316.
64. Sinigaglia, F., Takacs, B. & Romagnoli, P. (1992) Abstracts of the 8th Int. Congr. Immunol., Budapest 619.
65. Gleichmann, E., Kubicka-Muranyi, M., Griem, P. & Goebel, C. (1992) Br. J. Rheumatol. **31**, 45.
66. Uetrecht, J. P. (1992) Drug Metabolism Rev. **24**, 299-366.
67. Rubin, R. L. (1989) in Autoimmunity and Toxicology, eds. Kammüller, M. E., Bloksma, N. & Seinen, W. (Elsevier Science, Amsterdam), pp. 119-150.
68. Uetrecht, J. P. (1988) Chem. Res. Toxicol. **1**, 133-143.
69. Kubicka-Muranyi, M., Goebels, R., Goebel, C., Uetrecht, J. & Gleichmann, E. (1993) Toxicol. Appl. Pharmacol. **122**, 88-94.

